# Effects of High-Intensity Aquatic or Bicycling Training in Athletes with Unilateral Patellofemoral Pain Syndrome

**DOI:** 10.3390/ijerph19084675

**Published:** 2022-04-13

**Authors:** Bin Fang, Yong-hwan Kim, Moon-young Choi

**Affiliations:** 1 College of Physical Education, Luoyang Normal University, Luoyang 471934, China; fangbin@lynu.edu.cn; 2 Department of Physical Education, Gangneung-Wonju National University, Gangneung 25457, Korea; yhkim@gwnu.ac.kr; 3 Department of Sports Science Convergence, Dongguk University, Seoul 04620, Korea

**Keywords:** patellofemoral pain syndrome, training, bicycle, pain, muscle strength, physical fitness

## Abstract

Patellofemoral pain syndrome (PFPS) is one of the most common overuse injuries experienced by athletes. It is characterized by pain and functional deficits that lead to decreased performance, thereby limiting sports activity. Therefore, optimal training interventions are required to improve physical fitness and function while minimizing pain due to PFPS. This study aimed to compare and analyze the effects of high-intensity aquatic training (AT) and bicycling training (BT) in male athletes with PFPS. Fifty-four athletes with PFPS were divided into AT and BT intervention groups. Intervention training was conducted three times per week for 8 weeks. Cardiorespiratory fitness was evaluated using the graded exercise test (GXT) based on peak oxygen uptake (VO_2_ peak), and anaerobic threshold. For the knee strength test, extension and flexion were performed and measured using isokinetic equipment. One-leg hop tests and the Y-balance test (YBT) were performed to evaluate dynamic balance, and the International Knee Documentation Committee (IKDC) scoring system was used for subjective knee evaluation. The GXT, YBT, and IKDC scores were reported according to the group and duration of the intervention. After training, VO_2_ peak, YBT, knee extension strength, and IKDC score improved significantly in both the AT and BT groups compared with the pre-training values. Furthermore, the AT group exhibited significant improvement compared with the BT group. We demonstrated that AT and BT effectively improved the symptoms and muscle strength of athletes with PFPS who were only able to engage in limited high-intensity field training. AT produced a modestly better effect than BT.

## 1. Introduction

Patellofemoral pain syndrome (PFPS) is a term used to describe the pathological abnormalities associated with anterior knee pain [1]; it is one of the most common overuse injuries experienced by athletes involved in various sports [2]. PFPS accounts for approximately 25% of all knee injuries diagnosed in sports medicine clinics, and approximately 70% have been reported in adolescents and young athletes aged 16–25 years [3]. In particular, PFPS is very common in athletes participating in sports that require repetitive cutting, pivoting, and jumping, such as soccer, basketball, and volleyball [4]. The main symptom is pain around the patella, at the front of the knee, which intensifies with squatting, prolonged sitting, kneeling, or using force to bend and straighten the knee [5]. PFPS is accompanied by pain and functional deficits, which can lead to decreased performance and limit sports activity in athletes [1].

The etiology of PFPS includes overuse and biomechanical changes, such as increased Q-angle, quadricep weakness, joint laxity, and patellar instability, as well as chondral and osteochondral damage [6]. Among these factors, quadricep muscle strength is reported to be closely related to the occurrence of PFPS [7]. Athletes with PFPS exhibit atrophy and inhibition of the quadriceps compared with those of healthy athletes, possibly leading to a decrease in the peak torque of quadricep muscle strength [8]. Moreover, the imbalance in power between the vastus medialis oblique and vastus lateralis comprising the quadricep muscles negatively affects lateral patellar tracking [9]. Besier et al. [10] observed that patients with PFPS exhibited greater co-contraction of the quadricep and hamstring muscles during heel strike when walking and running compared to individuals without symptoms. Therefore, it is suggested that relatively low stimulation of the quadricep muscles causes knee pain, along with increased joint contact force and joint stress. As a result, athletes with PFPS experience pain and decreased knee joint function.

However, despite the pain and functional decline caused by patellofemoral pain, many athletes still participate in sports activities and are required to engage in high-intensity training to achieve maximum performance during the sporting season [11]. Specifically, in sports such as soccer and basketball that require repeated high-intensity sprints, rapid changes of direction, and jump-and-landing movements, training programs focus on high-intensity cardiorespiratory fitness (CRF) and skill training that reflect play characteristics [12,13]. Although it is difficult for athletes with PFPS to perform these high-intensity exercises completely, participation in high-intensity training is unavoidable, particularly for athletes participating in competitive sports, who are required to maintain optimal performance [14].

By reducing weight-bearing, aquatic training (AT) is an effective strategy for improving physical ability, thereby improving pain without exacerbating pathological stress [15]. Due to the mechanical properties of water, training performed in an aquatic environment is safer than that performed in a terrestrial environment, as it exerts less weight-bearing joint stress. Therefore, even athletes with PFPS can effectively perform high-intensity aquatic physical training [16]. Previous studies involving athletes with PFPS have primarily focused on improving the strength and functional movement of specific muscles related to the pain symptoms [4,17,18]. The application of such rehabilitation-oriented training programs has limitations regarding improvement of the physiological and physical abilities required for actual sports activities.

Therefore, this study aimed to determine whether high-intensity training in an aquatic environment can improve physical ability and muscle function while minimizing pain in athletes with PFPS. We analyzed the results of CRF, isokinetic knee strength, functional hop, Y-balance test (YBT), and the International Knee Documentation Committee (IKDC) subjective knee score between the aquatic training (AT) and stationary bicycle training (BT) groups.

## 2. Materials and Methods

### 2.1. Experimental Design

For this study, we recruited patients diagnosed with PFPS from orthopedic or rehabilitation medicine specialists through a bulletin board. Participants participated voluntarily and submitted written informed consent. This study complied with the Declaration of Helsinki and was approved by the Institutional Review Board of Gangneung-Wonju University (approval number: GWNU IRB 2021-13).

We divided the participants into two groups and conducted AT and BT for 8 weeks. Isokinetic muscle strength, hop test, dynamic balance test, and a subjective knee evaluation questionnaire were conducted pre- and post-training.

### 2.2. Participants

The patients who visited the sports rehabilitation center were male university athletes (age: 19–24 years). Participants were diagnosed after comprehensive radiological and physical examinations, and evaluation questionnaires. The exclusion criteria were a history of past injury or surgery (*n* = 5) and intra-knee injury confirmed by radiography findings such as meniscus tear or ligament injury (*n* = 8). Six participants who discontinued treatment or moved to another clinic during the trial period were also excluded. The final analysis included 54 patients (AT group, *n* = 27; BT group, *n* = 27). The participants were involved in soccer (*n* = 16), basketball (*n* = 8), sprinting (*n* = 3), badminton (*n* = 7), tennis (*n* = 2), taekwondo (*n* = 4), wrestling (*n* = 3), judo (*n* = 2), baseball (*n* = 5), and handball (*n* = 4). The AT and BT assignments were decided after discussion with the participants.

### 2.3. Graded Exercise Test

The graded exercise test (GXT) measures peak oxygen uptake (VO_2_ peak), anaerobic threshold, and heart rate (HR) recovery by a gradual stepwise increase in exercise intensity. The GXT was performed with a motor-driven treadmill and gas analyzer (Vmax229, Sensormedics Co., Yorba Linda, CA, USA) using the stepwise Bruce protocol; the speed and inclination of the treadmill were gradually increased every 3 min [19].

The benefit, purpose, procedure, and risks associated with the test were fully explained to the participants. The test was performed after obtaining written informed consent. To assess possible cardiac risks and ensure safety, an electrocardiogram analyzer (Case8000, GE Marquette Co., Milwaukee, WI, USA) was attached to enable continuous monitoring during the examination. Blood pressure was checked each minute during the test and the rating of perceived exertion (RPE) was recorded every 3 min. The test was conducted to measure the maximum capacity, but was terminated if electrocardiographic abnormalities were observed, or at the request of the participant [20]. The VO_2_ peak was considered the maximum value when the respiratory exchange ratio reached 1.10 or higher during the test.

The anaerobic threshold was analyzed using the ventilation volume, VO_2_, and VCO_2_ results were recorded every 10 s during the exercise test. The anaerobic threshold was set as the point at which the ventilation volume and VO_2_ rapidly increased. Finally, the HR recovery rate was calculated using the maximum HR during the test and the HR recorded at 1 min of recovery time [21].

HR recovery 1 min = ((HRmax − HR recovery 1 min)/HRmax) × 100

### 2.4. Knee Muscle Strength Test

**Figure 1:**
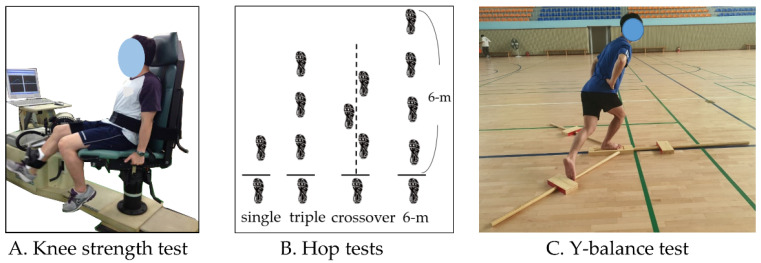
Lower extremity functional tests; (**A**) knee strength test using isokinetic device; (**B**) one-leg hop tests; (**C**) Y-balance test for dynamic balance.

Knee muscle strength was measured using an isokinetic dynamometer (Humac Norm, CSMi, Stoughton, MA, USA) to determine the isokinetic strength of the extensor and flexor muscles of the knee joint (Figure 1A). An isokinetic dynamometer resists mechanically applied force and measures muscle strength at a computer-controlled rate [22]. The test was performed at an angular velocity of 60°/s to measure the maximal muscle strength. The participant was placed in a sitting position with their back against the examination chair, and the anatomical axis of the knee (lateral epicondyle of the femur) was aligned with the axis of the dynamometer. Measurements were performed using concentric contractions of the knee extensor and flexor muscles.

To familiarize the participants with the machine, verbal explanations and demonstrations were conducted to enable full understanding of the test method; several practice exercises were conducted before the actual test. The range of motion of the knee for examination was set at 0° (extension) to 90° (flexion). The starting position was with the knee flexed at 90°, and the participant was instructed to perform knee extension and flexion with maximum effort in accordance with the examiner’s verbal command; the test was repeated four times. The peak torque (Nm) was recorded for the analysis.

Because muscle strength is affected by body weight, the relative value was obtained by dividing the absolute value by the body weight. In addition, to evaluate the hamstring to quadriceps ratio (H:Q ratio), the ratio of flexion and extension strength was analyzed [23].

H:Q ratio = (Flexion strength, Nm/kg/Extension strength, Nm/kg) × 100

### 2.5. Hop Tests

One-leg hop tests were performed to evaluate the functional performance of the lower limb [24]. Four tests were included: single hop, triple hop, crossover hop, and 6 m timed hop (Figure 1B). To prevent injury, the participant performed adequate warm-up exercises preceding the examination. The examiner demonstrated the procedure and allowed the participant to practice to gain familiarity with the examination method. The healthy leg was first examined, followed by the injured leg.

In the single-leg hop test, participants stood on one leg, jumped as far as possible with maximum effort, and landed on the same foot. For the triple-hop test, the participant performed three consecutive hops with one leg as far as possible in a straight line. In the crossover hop test, the participant hopped on one leg three times alternating to the left and right of the centerline as far as possible. In the 6 m timed hop test, the participant stood on one leg and moved a distance of 6 m by performing a series of consecutive single-leg hops as quickly as possible, without losing balance. The single, triple, and crossover hop tests measured the total hop distance, whereas for the 6 m timed hop test, a stopwatch was used to measure the time taken to complete the task. The test was performed twice, and the higher value was recorded and used for the analysis.

### 2.6. Y-Balance Tests

YBT equipment (Y-Balance Test ^TM^, Cerder Park, TX, USA) was used to evaluate the dynamic balance related to postural control (Figure 1C). The YBT is a dynamic test that requires lower extremity stability, flexibility, and proprioception [25,26]. The examiner provided the participant with sufficient time to practice after demonstrating the examination posture and movement. The participant placed the foot of the affected leg on the center footplate and raised the contralateral leg from the floor to stand on one leg. The participant maintained a single-leg stance, and YBT was performed by extending the contralateral leg as far as possible in anterior, posteromedial, and posterolateral directions. If the balance was lost, or the foot of the reaching limb touched the floor, it was considered a failure. After measuring each direction twice, the higher score was used for the analysis. The leg length of each participant was determined by measuring the distance between the anterior superior iliac spine of the pelvis and the medial malleolus of the distal tibia, using a tape measure. The total score for the three directions was calculated as follows:

YBY total score = [(sum of the three reach directions)/(limb length × 3)] × 100

### 2.7. Subjective Knee Score

The subjective knee score was measured using the subjective knee evaluation form distributed by the International Knee Documentation Committee (IKDC) [27]. The IKDC questionnaire is widely used to subjectively evaluate the condition of the knee, and the version used in this study exhibited excellent validity [28,29], with an intraclass correlation coefficient of 0.94 and Cronbach’s alpha of 0.91. The questionnaire comprised 18 items and evaluated pain, stiffness, swelling, locking/catching, and giving way in the last few days. Subjective knee function and sports participation rate were determined as perceived by the level of sports activity, going up and down stairs, kneeling, squatting, flexing the knee, sitting, running, jumping, and starting and stopping quickly. The total score was obtained by summing the scores for each item. A score of 100 indicated no knee symptoms or functional limitations, with no restriction on sports or daily activities, whereas 0 indicated the worst knee condition. The IKDC score was calculated as follows:

IKDC score = (sum of items/maximum possible score) × 100

### 2.8. Training Program

#### 2.8.1. Aquatic Training

**Figure 2:**
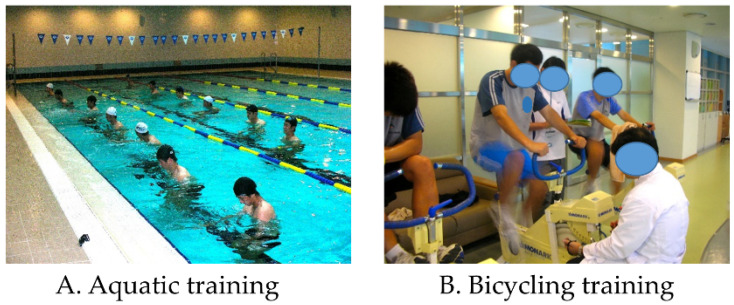
Aquatic training and bicycling training intervention.

The AT program was conducted three times per week for 8 weeks using a high-intensity interval training (HIIT) program in water [30] (Figure 2A). The aquatic HIIT program comprised eight routines in which four water aerobic exercises (stationary running, cross-country skiing, jumping jacks, and frontal kick to 90°) were performed in duplicate. Each set included 2 min of vigorous exercise, followed by 2 min of interval recovery. Therefore, the total training time was 32 min. All lower body movements were performed simultaneously with bilateral arm push pulls. For the target HR during the AT program, 75–85% of HR max was applied by the Karvonen formula using the HR obtained from the GXT [16,31]. Monitoring during training was assessed with the Borg’s rating of perceived exertion (RPE) scale using verbal scales [32].

The examiner trained the participants regarding the standard guidelines of the RPE scale to enable participants to verbally express their perceived level of effort as accurately as possible. In addition, sufficient practice was performed before training to familiarize the participants with the feelings corresponding to minimum and maximum effort. The participants performed water aerobic exercise with an RPE intensity of 17, corresponding to the verbal scale of “very hard” with maximum effort for 2 min after the trainer’s start signal. Active recovery between each vigorous exercise was performed for 2 min at an intensity of RPE 9, corresponding to the verbal scale of “very light.” Additionally, the RPE for each training session was monitored along with the participant’s actual HR obtained using an electronic heart-rate-monitoring device (Polar H10, Polar Electro, Bethpage, NY, USA).

#### 2.8.2. Bicycling Training

The BT program was conducted three times per week for 8 weeks by applying the repeated Wingate sprint protocol previously described [33] (Figure 2B). The repeated Wingate sprint protocol is a bicycling sprint HIIT method that involves close to “all out” effort for 30 s. A friction-loaded bicycle ergometer (Monark model 864 Crescent AB, Varberg, Sweden) was used for the training. The height of the saddle was adjusted based on the individual body structure of the participants [34]. The height of the saddle was adjusted such that when the participant sat on the saddle and extended one leg as far as possible, it was approximately 25°.

Participants performed warm-up bicycling at 60 rpm for 10 min under a load of 1 kp corresponding to 50 W. Individual load (0.075 × kg body mass^−1^) according to the participant’s body weight was applied along with a starting signal; the participant was verbally encouraged to pedal as quickly as possible for 30 s. After a 30 s bicycling sprint, the participant took an active rest, maintaining 60 rpm for 2 min at a load of 1 kp. During the last 5 s of the active rest period, the participant again achieved 100 rpm or more, and the 30 s bicycling sprint was repeated. During the entire training, the participants performed six sets of 30 s bicycling sprints at 2 min intervals. After this exercise, cool-down was performed for 10 min at 60 rpm with a 1 kp load.

#### 2.8.3. Muscle Strength Training

In addition to the intervention training, a strength training program was conducted, which was performed in the same manner in the AT and BT groups. The strength training program involved weight training that focused on strengthening the muscles, and was conducted according to the recommendations of the American College of Sports Medicine, and 12 repetitions of three sets were performed at 80% of 1RM [35]. Weight training included leg extension, leg curl, leg press, hip abduction, inner thigh, shoulder press, chest press, butterfly, lat pull-down, long pull, arm curl, and abdominal machine use. Considering the pathological characteristics of PFPS, special emphasis was placed on maintaining normal knee alignment when performing knee extension using the leg-extension machine. In addition, if pain was present in the symptomatic leg during strength training, more force was applied to the contralateral leg to facilitate as much pain-free performance as possible.

Athletes participating in the training from both groups were monitored at the center, and were instructed not to participate in sports team training or sports skills training for 8 weeks.

### 2.9. Statistical Analysis

G*power software (G*power 3.1, University of Düsseldorf, Düsseldorf, Germany) was used for sample size calculation. The conditions were as follows: effect size f = 0.25; α error probability = 0.05; and power (1-β error probability) = 0.95. The data were analyzed using IBM SPSS Statistics for Windows (version 25.0; IBM Corp., Armonk, NY, USA). Continuous variables are presented as means and standard deviations and categorical variables as numbers and percentages. Normality tests were performed using the Kolmogorov–Smirnov and Shapiro–Wilk tests, and nonparametric analyses were performed because the main variables analyzed in this study were not normally distributed. The Wilcoxon test was used for pre- and post- intra-group comparisons, whereas the Mann–Whitney U test was used for between-group comparisons. Repeated two-way analysis of variance was performed to evaluate the interaction between time and group. The significance level was set at *p* < 0.05.

## 3. Results

### 3.1. General Characteristics of Participants

Participants were classified according to the intervention group, and Table 1 summarizes the general characteristics of the participants. There were no significant differences in age, height, weight, or body mass index (BMI) between the AT and BT groups.

### 3.2. Cardiorespiratory Fitness

Table 2 shows changes in the GXT before and after training. Changes in VO_2_ peak, anaerobic threshold, and HR recovery significantly increased in both the AT and BT groups. After training, there were significant differences between the AT and BT groups for all three variables, and the interaction between time and group was significant.

### 3.3. Isokinetic Knee Strength

Table 3 presents the changes in isokinetic knee strength. Knee extension strength in both the AT and BT groups increased significantly after training, but there were no significant between-group differences. Additionally, no significant differences were observed in flexion strength after training between the AT and BT groups. The H:Q ratios decreased significantly after training in both the AT and BT groups compared to the pre-training values; however, there was no significant difference between the groups.

### 3.4. Hop Tests

Table 4 summarizes the hop test results. Single, triple, crossover, and 6 m hop tests increased significantly after training compared to before training in both the AT and BT groups. However, there were no significant between-group differences after training, and the interactions according to time and group were not significant.

### 3.5. Y-Balance Test

Table 5 lists the YBT results. The anterior, posteromedial, and posterolateral reach distances and the total scores were significantly higher after training than before training in both AT and BT groups. In the between-group comparison after training, the YBT of the AT group was significantly higher than that of the BT group, and the interaction was also significant.

### 3.6. Subjective Knee Score

Table 6 shows the results of the subjective knee scores evaluated using the IKDC. Both AT and BT groups exhibited a significant increase in IKDC score after training. The between-group comparison revealed that the AT group achieved a significantly higher score after training than the BT group.

## 4. Discussion

PFPS is caused by several factors, the most common being overuse, patellar–femoral misalignment, and muscle imbalance [4,36]. For athletes with PFPS, it is important to design a safe high-intensity conditioning program because rehabilitation-focused training has limitations for athletes aiming to improve and maintain high-level physiological and physical performance.

Training in water enables high-intensity training for athletes with PFPS because it reduces weight bearing and shock while providing adequate resistance to improve CRF and muscle function [37]. Previous studies have reported that AT relieves pain and promotes recovery, suggesting that it may complement ground training to improve aerobic performance, speed, strength, and power [38,39,40]. Another training method, BT, has been proposed as a land-based training intervention to reduce pain by minimizing the stress caused by weight bearing whilst improving CRF and muscle function because it is performed while sitting on a stationary bicycle [41,42,43].

The GXT results of this study revealed that the VO_2_ peak, anaerobic threshold, and HR recovery improved significantly after training in both the AT and BT groups. However, after training, in the time and group interaction, the AT group exhibited significantly better results for all GXT variables than the BT group. These results are likely due to the specific physiological responses of the body to the aquatic environment. Previous studies have reported that training in an aquatic environment significantly increases the efficiency of cardiac output, oxygenation to fatigued muscles, and the delivery of nutrients and hormones, compared with land-based training [44,45]. Hydrostatic pressure is applied when a person’s body is submerged in water. This pressure is directly proportional to the density of the liquid, its gravity, and the depth at which the body is submerged [46]. The deeper the immersion in water, the greater is the pressure on the body. In our study, participants in the AT group performed high-intensity training with their body immersed in water to the depth of their chest. Therefore, whereas BT is focused on high-intensity load and fatigue only on the lower body, AT provides greater load and stimulation throughout the body. Owing to these differences, a greater cardiac metabolic response was induced in the AT group than that in the BT group.

Previous studies have demonstrated that improved performance in patients with PFPS is associated with maximal quadricep muscle strength [47,48]. Decreased quadricep muscle strength is considered to reflect pain in patients with PFPS [49]. Therefore, the recovery of quadricep muscle function is crucial for improving the symptoms and functions caused by PFPS. Regarding the isokinetic knee strength, both the AT and BT groups significantly improved extension strength after training. Moreover, the ratio of extensor to flexor strength improved. These results suggest that 8 weeks of AT and BT are effective interventions for improving quadricep muscle strength in athletes with PFPS. In previous studies, several authors have reported that cycle-based HIIT significantly improved isokinetic knee strength [50,51]. However, considering that BT generates repetitive flexion and extension in the knee, along with the load applied through pedaling, there is the possibility of negatively impacting athletes with PFPS. Meanwhile, AT can provide sufficient load to induce muscle contraction and improve muscle strength, whilst almost eliminating weight-bearing stress to the knee [40]. Therefore, when BT is limited owing to pain caused by PFPS, AT may be effective.

Functional hop tests are used to measure lower extremity functions and are often used to assess recovery after knee injuries [52]. The results of the functional hop tests in our study were significantly improved after training in both groups. Previous studies revealed a significant correlation between reduced hop test performance and increased H:Q ratio [53]. In our study, both the AT and BT groups showed a significant reduction in the H:Q ratio with an increase in knee extension strength, and with improvements in the performance of the functional hop tests.

Dynamic balance is necessary to complete activities in daily life, as well as during sports activities, and is essential for maintaining balance when performing actions such as running, jumping, and landing [54,55]. Several studies have reported that the YBT is useful for measuring dynamic balance and motor function, with high validity and reliability [56,57]. In our study, the YBT for the anterior, posteromedial, and posterolateral distances and total scores improved significantly after training in both the AT and BT groups. However, the between-group comparison revealed that the AT group achieved a more significant improvement than the BT group. Training in water can cause instability; this can alter information in the somatosensory system as a result of weight loss due to buoyancy. Water turbulence can also be an additional balance stimulus that increases the challenge of a task [58,59]. Bento et al. [60] reported that continuous perturbation due to water turbulence activated the neuromuscular system of the ankle and knee joints to restore balance throughout the training sessions. In addition, it is known that the impairment of dynamic balance control in patients with severe knee pain is due to a lack of neuromuscular control resulting from changes in somatosensory input [61,62]. Therefore, it is believed that the hydrostatic properties of water positively affect dynamic balance related to neuromuscular control and somatosensory input [46].

In previous studies, various questionnaires have been proposed to facilitate the functional diagnosis of patients with PFPS and to characterize functional limitations [63,64]. The results of these functional measures can contribute to the comprehensive evaluation of therapeutic intervention strategies. Because the subjective scale evaluates functional activities such as climbing stairs, sitting, standing, and squatting, it is possible to estimate the level of effectiveness of intervention programs and the level of functioning required to participate in sports activities [65]. Among several functional measures, the IKDC scale is commonly used in orthopedics and sports medicine to evaluate subjective knee function. The IKDC scale comprises subdivided multiple-choice questions on symptoms, including knee pain, stiffness, instability, knee function, and participation in sports activities, and has been reported to exhibit high reliability, even in patients with PFPS [66]. In this study, the IKDC score improved significantly after training in both groups, and the AT group improved to a greater extent than the BT group. This difference means that subjective symptoms improved more in the AT group than in the BT group. Underwater training has been reported to relieve pain, reduce swelling, increase blood circulation, and promote recovery, because it reduces weight bearing due to buoyancy while providing features such as water pressure and turbulence [37,46]. Therefore, the advantage of aquatic training appears to include improved recovery and fatigue after training.

In sports practice, the aquatic environment is a less common condition than bicycling, but it should be more widely applied to improve the pain and physical condition of athletes with PFPS. This study has some limitations. Because the AT or BT assignment was not randomized, it is possible that participants’ preferences influenced the outcomes. Additionally, the functional improvement due to natural healing could not be determined because no control group was established. We instructed all athletes to refrain from training outside the center program, but there were limits to which private training could be controlled. Moreover, there would have been ethical issues associated with asking athletes who attended the clinic for treatment to deliberately refrain from intervention for the purpose of research. In addition, the sample size was relatively small, and the study was conducted at a single center. In aquatic therapy or exercise, the temperature of the water is an important consideration. However, since this study was conducted in a general swimming pool, not a treatment-only facility, the temperature of the water could not be controlled [67]. We provided an individualized program that took into account the tolerable pain range. However, the detail of the program was limited because the pain severity of the patients was very diverse. Therefore, future research is required with a design that compensates for these limitations and that investigates various training programs aimed at preventing injuries and improving performance, thereby contributing to the development of sports training.

## 5. Conclusions

Both AT and BT significantly improved all GXT results after 8 weeks, including isokinetic knee extension strength, functional hop tests, YBT, and the IKDC score after training. However, analysis according to time and group revealed that AT provided greater improvement in GXT and YBT than BT. In addition, subjective satisfaction was significantly higher in the AT group. Therefore, for athletes who are restricted from high-intensity field training due to PFPS, AT and BT could be effective training interventions that can improve their symptoms and physical strength, with AT achieving modestly better results.

## Figures and Tables

**Table 1:** General Characteristics of Participants.

Variables	AT (*n* = 27)	BT (*n* = 27)	t or χ^2^	*p*-Values
Age, years	20.8 ± 1.7	20.9 ± 1.9	0.031	0.976
Height, cm	179.1 ± 6.0	178.9 ± 6.5	0.823	0.420
Weight, kg	75.8 ± 5.5	76.3 ± 6.7	−1.027	0.316
BMI, kg/m^2^	23.6 ± 1.8	23.8 ± 1.9	−2.239	0.210
Left/Right side	12/15	16/11	0.667	0.587
Dominant/Non-Dominant	10/17	12/15	0.307	0.782

Abbreviations: AT, aquatic training; BT, bicycling training; BMI, body mass index.

**Table 2:** Graded exercise test.

Variables	Group	Pre-Training	Post-Training	Difference (%)	*p*-Values	Time × Group *p*-Values
VO_2_ peak, mL/kg/min	AT	43.2 ± 6.3	56.1 ± 8.4	29.9	<0.001	0.021
	BT	44.7 ± 7.1	53.3 ± 8.3	19.2	<0.001	
	*p*-values	0.423	0.015			
Anaerobic threshold, %	AT	67.2 ± 9.3	74.5 ± 6.7	10.9	<0.001	0.034
	BT	66.1 ± 8.9	70.3 ± 7.1	6.4	0.005	
	*p*-values	0.510	0.020			
HR recovery 1 min, %	AT	56.4 ± 6.6	69.1 ± 10.7	22.5	<0.001	0.011
	BT	56.3 ± 7.9	66.3 ± 11.1	17.8	0.009	
	*p*-values	0.399	0.010			

*p* < 0.05; Abbreviations: AT, aquatic training; BT, bicycling training.

**Table 3:** Isokinetic knee strength test (60°/s).

Variables	Group	Pre-Training	Post-Training	Difference (%)	*p*-Values	Time × Group *p*-Values
Extension strength, Nm/kg, %	AT	255.2 ± 45.1	305.6 ± 48.4	19.7	0.010	0.129
	BT	258.9 ± 50.0	295.9 ± 52.7	14.3	0.012	
	*p*-values	0.404	0.332			
Flexion strength, Nm/kg, %	AT	172.3 ± 21.0	182.5 ± 25.6	5.9	0.106	0.230
	BT	170.3 ± 29.1	179.8 ± 31.3	5.6	0.216	
	*p*-values	0.215	0.320			
H:Q ratio	AT	66.2 ± 16.0	59.7 ± 15.6	−11.5	0.004	0.210
	BT	65.8 ± 19.1	60.8 ± 11.3	−7.6	0.007	
	*p*-values	0.469	0.221			

*p* < 0.05; Abbreviations: AT, aquatic training; BT, bicycling training; Nm, Newton meter; H:Q ratio, Hamstring: Quadriceps ratio.

**Table 4:** Results of hop tests for bicycling and aquatic training groups.

Variables	Group	Pre-Training	Post-Training	Difference (%)	*p*-Values	Time × Group *p*-Values
Single, cm	AT	146.1 ± 20.3	169.3 ± 19.4	15.9	<0.001	0.515
	BT	147.4 ± 19.3	170.2 ± 23.3	15.5	<0.001	
	*p*-values	0.521	0.215			
Triple, cm	AT	460.3 ± 41.5	493.3 ± 42.9	7.2	<0.001	0.611
	BT	454.3 ± 43.1	486.4 ± 54.3	7.1	<0.001	
	*p*-values	0.419	0.318			
Crossover, cm	AT	412.7 ± 35.7	444.9 ± 31.4	7.8	<0.001	0.318
	BT	416.2 ± 37.3	454.4 ± 33.5	9.2	<0.001	
	*p*-values	0.514	0.325			
6 m, s	AT	2.19 ± 0.17	2.10 ± 0.13	−4.1	0.011	0.119
	BT	2.21 ± 0.16	2.07 ± 0.11	−6.3	0.018	
	*p*-values	0.128	0.498			

*p* < 0.05; Abbreviations: AT, aquatic training; BT, bicycling training.

**Table 5:** Y-balance test results of aquatic and bicycling training groups.

Variables	Group	Pre-Training	Post-Training	Difference (%)	*p*-Values	Time × Group *p*-Values
Anterior	AT	54.3 ± 15.3	70.5 ± 17.1	29.8	0.006	0.014
	BT	56.1 ± 17.0	63.3 ± 19.4	12.8	0.012	
	*p*-values	0.621	0.008			
Posteromedial	AT	66.1 ± 19.6	78.7 ± 21.6	19.1	0.021	0.016
	BT	65.3 ± 21.0	70.6 ± 23.4	8.1	0.010	
	*p*-values	0.204	0.006			
Posterolateral	AT	65.9 ± 25.9	79.6 ± 22.7	20.8	<0.001	0.011
	BT	62.5 ± 26.1	72.4 ± 23.8	15.8	0.005	
	*p*-values	0.634	0.012			
Total	AT	73.1 ± 18.3	85.4 ± 15.1	16.8	<0.001	0.035
	BT	71.7 ± 20.1	79.7 ± 17.6	11.2	0.003	
	*p*-values	0.540	0.015			

*p* < 0.05; Abbreviations: AT, aquatic training; BT, bicycling training.

**Table 6:** International Knee Documentation Committee scores of bicycling and aquatic training groups.

Variables	Group	Pre-Training	Post-Training	Difference (%)	*p*-Values	Time × Group *p*-Values
IKDC score	AT	65.2 ± 13.9	88.4 ± 13.3	35.6	<0.001	0.019
	BT	67.6 ± 15.0	85.8 ± 12.1	26.9	0.004	
	*p*-values	0.241	0.022			

*p* < 0.05; Abbreviations: AT, aquatic training; BT, bicycling training; IKDC, International Knee Documentation Committee.

## Data Availability

The data are not publicly available because of privacy or ethics.
